# Smoke from regional wildfires alters lake ecology

**DOI:** 10.1038/s41598-021-89926-6

**Published:** 2021-05-25

**Authors:** Facundo Scordo, Sudeep Chandra, Erin Suenaga, Suzanne J. Kelson, Joshua Culpepper, Lucia Scaff, Flavia Tromboni, Timothy J. Caldwell, Carina Seitz, Juan E. Fiorenza, Craig E. Williamson, Steven Sadro, Kevin C. Rose, Simon R. Poulson

**Affiliations:** 1 Global Water Center, Department of Biology, University of Nevada, Reno, Reno, NV USA; 2 Division of Hydrologic Sciences, Desert Research Institute, Reno, NV USA; 3 Global Water Futures, CFREF, University of Saskatchewan, Saskatoon, SK Canada; 4 Consejo Nacional de Investigaciones Científicas y Tecnológicas (CONICET), Universidad de Buenos Aires (UBA), Instituto de Investigaciones Fisiológicas y Ecológicas Vinculadas a la Agricultura, (IFEVA), Buenos Aires, Argentina; 5 Facultad de Agronomía, Departamento de Métodos Cuantitativos y Sistemas de Información, Universidad de Buenos Aires, Buenos Aires, Argentina; 6 Department of Biology, Miami University, Oxford, OH USA; 7 Department of Environmental Science and Policy, University of California, Davis, Davis, CA USA; 8 Department of Biological Sciences, Rensselaer Polytechnic Institute, Troy, New York, NY USA; 9 Department of Geological Sciences and Engineering, University of Nevada, Reno, Reno, NV USA

**Keywords:** Ecology, Environmental sciences, Limnology, Natural hazards

## Abstract

Wildfire smoke often covers areas larger than the burned area, yet the impacts of smoke on nearby aquatic ecosystems are understudied. In the summer of 2018, wildfire smoke covered Castle Lake (California, USA) for 55 days. We quantified the influence of smoke on the lake by comparing the physics, chemistry, productivity, and animal ecology in the prior four years (2014–2017) to the smoke year (2018). Smoke reduced incident ultraviolet-B (UV-B) radiation by 31% and photosynthetically active radiation (PAR) by 11%. Similarly, underwater UV-B and PAR decreased by 65 and 44%, respectively, and lake heat content decreased by 7%. While the nutrient limitation of primary production did not change, shallow production in the offshore habitat increased by 109%, likely due to a release from photoinhibition. In contrast, deep-water, primary production decreased and the deep-water peak in chlorophyll *a* did not develop, likely due to reduced PAR. Despite the structural changes in primary production, light, and temperature, we observed little significant change in zooplankton biomass, community composition, or migration pattern. Trout were absent from the littoral-benthic habitat during the smoke period. The duration and intensity of smoke influences light regimes, heat content, and productivity, with differing responses to consumers.

## Introduction

In the last 350 million years, wildfires have controlled land surface and atmospheric dynamics by removing underbrush, catalyzing new canopy growth, and transporting carbon and nutrients to distant locations by regional wind currents^1,2^. Anthropogenic-driven warming has increased the intensity and frequency of wildfire events worldwide^3^ and the duration of wildfire seasons has been extended in recent decades^2,4,5^. Unprecedented in contemporary history, multiple significant fires occurred across several countries during 2018, including Russia, Greece, the United Kingdom, Sweden, and Australia^6^. In 2018 in North America, fires in British Columbia burned more area than any year previously recorded^7^. At the time, this year was also the most destructive wildfire year ever recorded in California, USA, with a total of 7638 fires that burned an area of 794,438 ha^8^. Aquatic ecosystems in the western United States have a high risk of direct and indirect exposure to wildfires^9^, owing to the region’s sensitivity to increasing temperatures and relative humidity deficit^5,10^, the increase in people living and recreating in fire-prone landscapes^11–13^, and the accumulation of fuel from previously implemented fire suppression plans^4^. Despite growing concern of wildfires in western North America, the ecological effects of wildfires on aquatic ecosystems remain unclear.

**Figure 1:**
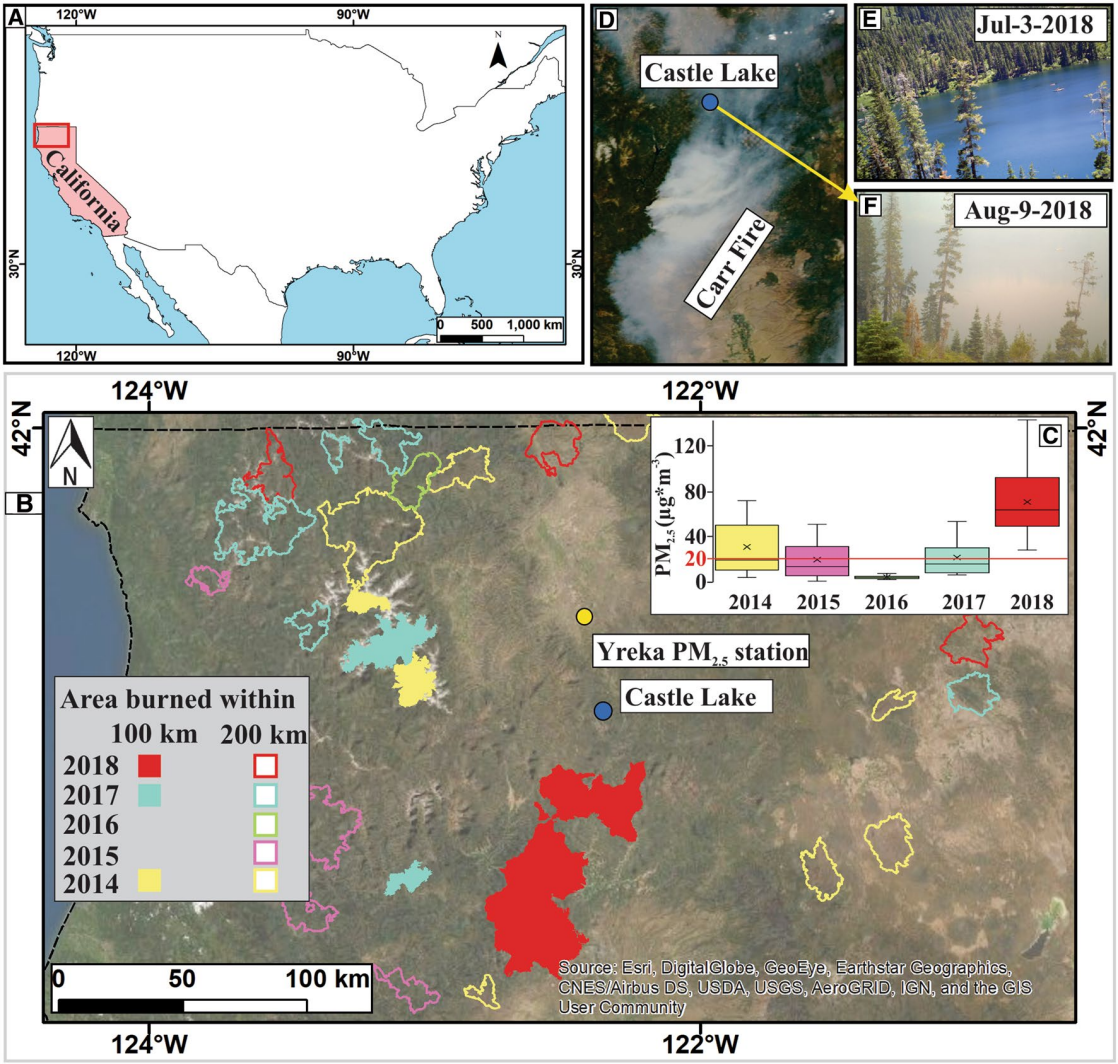
(**A**) Location of the study area in the state of California (USA). (**B**) Location of Castle Lake in northern California and the area burned (larger than 40 km^2^) that occurred during 2018, 2017, 2016, 2015, and 2014, within 100 km and 200 km radius from the lake. (**C**) Boxplot of daily concentration of particulate matter in the air (PM_2.5_) from July 18 to September 10 (concentrations above 20 µg*m^−3^ in fire prone areas is associated with wildfire smoke plumes). (**D**) The smoke plume from Carr Fire on August 9th, 2018 drifting down across the location of Castle Lake. Castle Lake on the 3rd of July 2018 without smoke (**E**), and on the 9th of August 2018 with smoke (**F**). Area burned polygons where obtained from MTBS^73^. Map was generated in ArcGIS 10.8.1^46^.

Wildfires can influence aquatic ecosystems both within a basin and via atmospheric connections of smoke emission^9^. Within a catchment, burned vegetation increases nutrients and particulate material in the soil, which is transported to lakes and rivers by overland flow and subsurface runoff^14–17^. Fires also emit smoke and particles that can be transported within or across watersheds affecting water bodies near and distant from the fire^9^. Wildfire smoke reduces light in a wavelength-selective manner that decreases the ratio between ultraviolet B radiation (UV-B) and photosynthetically active radiation (PAR)^18^. Additionally, smoke plumes deposit organic and inorganic particulate material on the water surface^19^, which can further reduce light penetration^15,20^. Ash deposition also releases ions, micronutrients, and macronutrients into freshwater ecosystems^21^ that stimulate primary productivity^19^. These shifts in water transparency and production likely influence ecosystem function, including changes in phytoplankton and zooplankton abundance and community composition, the vertical distribution of zooplankton, and UV-driven mortality rates of waterborne parasites^18,22–24^. Although these effects of wildfire smoke have been observed across different ecosystems, no study has examined how wildfire smoke simultaneously influences the physical, chemical, and biological characteristics within a single lake ecosystem. Untangling when smoke from wildfire influences the quality and type of light dynamics, the potential alteration to nutrient limitation for phytoplankton through fertilization of waters, the diversity of zooplankton community and thus potential for grazing on production, or changes to fish consumer behavior allows for a more robust and dynamic understanding of a lake’s response. Furthermore, due to the unexpected and dangerous nature of the wildfires, most studies of fire effects on lakes do not examine lake responses during fire events, instead, our understanding of wildfire effects often start after a fire is extinguished^9^. Research that analyzes responses of limnological variables while fires are occurring fill an important literature gap in the lake responses to fire. In this study, we examine the influence of wildfire smoke on light dynamics, heat content, production, and animal composition and behavior in Castle Lake, a subalpine lake in Northern California (USA). Six major fires occurred between July and September of 2018 within a 160 km radius of Castle Lake. (Fig. 1A, B, E, D, and Supplemental Material Sect. 1^8^). As a result, wildfire smoke covered the lake basin for 55 days between July and September of 2018, encompassing 60% of the productive, ice-free period.

## Materials and methods

Castle Lake (41′13″ N, 122′22″ W) is a subalpine, meso-oligotrophic, and dimictic lake with a surface area of 0.2 km^2^, a maximum depth of 35 m, and a mean depth of 11.4 m. The lake is ice-free for 135 days on average, from spring to fall. We compared the smoke condition of Castle Lake during the fire period of July to September of 2018 with previous four years (2014–2017). We used field notes and photos taken by a camera located in the watershed to identify days when smoke was present on the lake. We validated the photos by analyzing the concentration of fine (less than 2.5 μm diameter) particulate matter (PM_2.5_) in the air, which is a primary pollutant in wildfire smoke^25^. Concentration of PM_2.5_ higher than 20 μg*m^−3^ in fire prone areas is associated with wildfire smoke plumes^26^, while concentrations 35 μg*m^−3^ or higher reflect dense smoke conditions^27^. We obtained PM_2.5_ data from a nearby monitoring station located in the city of Yreka (https://www.epa.gov/outdoor-air-quality-data/download-daily-data).

To assess the influence of wildfire smoke on lake function, we compared data collected during the smoke season of 2018 to data collected in previous years (2014–2017). We chose to compare the smoke year of 2018 with multiple years rather than a single year to provide a conservative estimate of the influence of smoke conditions to lake function. Like other mountain lakes^28^, the ecology of Castle Lake exhibits strong interannual variation due to the timing of the spring thaw, ice-out date, and the amount of snowpack that accumulates in the basin, measured as snow water equivalent (SWE) (Supplemental Material Sect. 2). The four years prior to 2018 used in our comparison (2014–2017) encapsulated the total observed variability in the SWE (0–484 mm) and ice out date (February 20th to June 26th) in the last 20 years for this lake. These two factors have been previously shown to be important variables governing the heat content and primary production of Castle Lake^29–31^. The year 2018 was an average year in terms of hydroclimatic variables, as the ice out date was April 7th, and SWE was 135 mm (https://nsidc.org/data/g02158). This all-inclusive approach suggests that any novel patterns that we detected in 2018, relative to previously observed variability, are likely not related to unusual ice-out timing or snowpack that year, but rather to the unusual wildfire conditions.

To analyze the effect of smoke on the concentration of suspended sediment in the lake, we analyzed the concentration of particulate carbon (C) and nitrogen (N), and the C:N ratio in the composition of seston. We measured seston composition at 3 discrete depths in the epilimnion (0, 3 and 5 m depth; Supplemental Material Sect. 3). We collected particulate seston on precombusted 13 mm Whatman GF/F filters. We dried the filters at 60 °C for 24 h in an oven with acidified air to remove carbonates. The filters were then placed in tinfoil and analyzed for particulate carbon and nitrogen using an elemental analyzer (Eurovector EA3000) interfaces to a Micromass IsoPrime stable isotope ratio mass spectrometer. Here we present composite epilimnetic values of particulate carbon and nitrogen.

We evaluated how smoke altered the incoming solar radiation, water transparency to light and lake heat content. We calculated the midday (1 pm) incident UV-B (320 nm) and PAR (400–700 nm) radiation (300–1000 nm) above the surface of the lake using a Biospherical Instruments 2104P radiometer. We analyzed the seasonal pattern of the depth to which 1% of UV-B and PAR (as a percentage of subsurface radiation) penetrates the lake water column. We also analyzed the intensity of UV-B at 2 m deep, and PAR at 12.5 m deep. We used the radiometer to measure subsurface UV-B and PAR every ~ 0.1 m depth resolution. Finally, we used bathymetry data and weekly temperature profiles collected with the radiometer to calculate the lake heat content and the thermocline depth using the R package ‘rLakeAnalyzer’^32,33^.

Next, we analyzed depth-specific changes in net primary productivity, and the concentration of algal biomass (measured as chlorophyll *a*). We measured net primary productivity using an in situ ^14^C method^34^ from 13 discrete depths (Supplemental Material Sect. 3). In parallel with primary productivity experiments, we measured pheophytin-corrected chlorophyll *a* with methanol extraction and analyzed samples on a Turner 10-AU fluorometer^35^.

We collected zooplankton using a 12 L Schindler trap from the epilimnion (Supplemental Material Sect. 3) and preserved them in a Lugol's solution. Since zooplankton in Castle Lake typically express a strong diel vertical migration^36^, we collect day and night samples, and then estimated the extent of zooplankton diel vertical migration by subtracting the night from day time density of zooplankton (individuals*L^−1^). We identified zooplankton to genus and estimated zooplankton biomass (µg*L^−1^) using density-dry mass regressions determined previously for Castle Lake zooplankton^36^. We used the proportion of group-specific biomass (*Daphnia* sp., *Bosmina* sp., *Holopedium* sp., *Diacyclops* sp., *Diaptomus* sp.) as a measure of community composition. Our abundance and species composition values represent an average of day and night values.

Brook trout (*Salvelinus fontinalis*) and rainbow trout (*Oncorhynchus mykiss*) are the dominant fish consumer in Castle Lake, controlling plankton composition and productivity*.* We collected fishes using overnight (12 h) gill net sets (30 m long and 2 m high, 6–38 mm mesh size) from two locations in the littoral-benthic habitat (5 m and 2 m depth) at monthly intervals from June to October. Fish catch per unit effort (individuals*h^−1^) was used to estimate fish density for each species.

To understand whether smoke had an influence on the nutrient limitation of phytoplankton production, we compared the results of nutrient bioassays at the end of each summer from 2015 to 2018. In August of each year, we conducted 5-day bioassay experiments to assess nutrient limitation at the depth of the chlorophyll *a* maximum (15–20 m) and epilimnion (0, 3, and 5 m). Three replicates of 1L bottles were used for the following treatments: controls (C), nitrogen (N), phosphorus (P), and nitrogen plus phosphorus (N + P). In the nutrient-enriched treatments, we added 1 ml of 50 ppm of phosphorus (K_2_HPO_4_) and 300 ppm of nitrogen (NH_4_NO_3_) to each respective treatment^37,38^. We followed the hierarchical logic order presented in Maberly et al^39^ to determine the nutrient limiting phytoplankton growth: 1. P > C and N > C, both nutrients limiting; 2. P > C, P limitation, 3. N > C, N limitation, 4. NP > N or NP > P, Co-limitation; 5. P ≤ C and N ≤ C and NP ≤ N and NP ≤ P, no limitation.

We analyzed the differences among years in the proportions of days with PM_2.5_ higher than 20 μg*m^−3^ using a binomial generalized linear model (GLM) with “logit” as a link function. The years were placed as a fixed effect. We did not include the year 2016 in the binomial GLM since there were no smokey days during this year. The models were built in R using the "glm" function in the “stats” package^40^. We performed tests for the significance of the effects of the years in the models via the Wald statistic^41^. Multiple comparisons among years were performed with Tukey's HSD post hoc test using the "emmeans" package^42^.

We analyzed the association between PM_2.5_ (as a surrogate of smoke) and the different limnological variables using linear mixed-effects models (LMM). The PM_2.5_ was placed as a fixed effect and the dates as random effects. The models were built in R using “lme” function, in the “nlme” package^43^. LMM describes the linear association between two variables. However, we hypothesized that changes in some variables such as heat content, chemical and biological variables might lag from PM_2.5_ changes. Therefore, for each variable, we also calculated the mean, standard error, and 95% confidence interval for non-smoke years. Next, we determined if the data from the smoke-impacted 2018 fell within or outside of the 95% confidence interval of the non-smoke years. For calculating the 95% confidence interval we used a Student's t-distribution:

$$\underline {X} \pm t_{n - 1} \frac{SE}{{\sqrt n - 1}}$$

where $\underline {X}$ is the mean of the non-smoke years for a given variable, $SE$ is the standard error of the non-smoke years for the corresponding variable, n is the sample size, and $t_{n - 1}$ is the t-statistic corresponding to $1 - \frac{\alpha }{2}$ with n−1 degrees of freedom.

For the bioassay experiment, we used ANOVAs and pairwise Tukey tests to determine if treatments differed from the control in 2018, and in 2015–2017 (all years combined). To compare the zooplankton community composition data, we ran a permutational analysis of variance^44^ in R using the “vegan”^45^ package. All the analyses were performed in the statistical software R version 4.0.2^40^.

**Figure 2:**
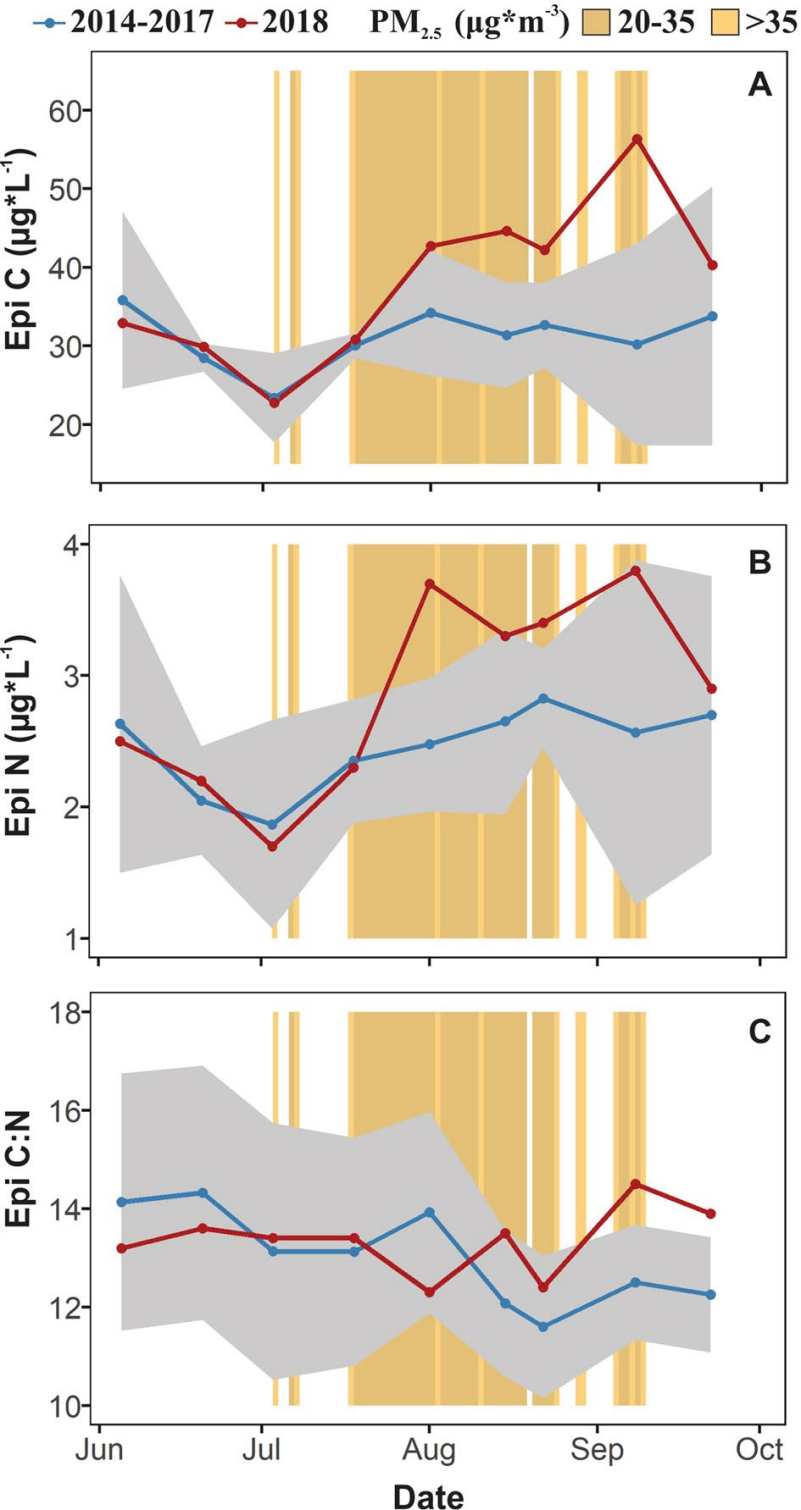
Summer seasonal pattern of the concentration of fine particulate matter (PM_2.5_ measured at Yreka station) in the air, and the concentration of particulate carbon (**A**), particulate nitrogen (**B**), and the carbon–nitrogen ratio (C:N) in the shallow waters of the lake (**C**). We compared the concentration of particulate carbon and nitrogen of the 2018 year with smoke (red) to the mean (± 95% confidence interval in gray) for non-smoke years from 2014 to 2017 (blue). Smoke from wildfires covered Castle Lake for 55 days between July and September of 2018. Orange background represent days with different smoke intensities in 2018 where PM_2.5_ in the range of 20–35 ug*m^−3^ represents lower smoke conditions and > 35 present substantially higher smoke conditions in the lake’s watershed**.** Research has documented elevated PM_2.5_ as a signal of wildfire smoke in the atmosphere.

**Figure 3:**
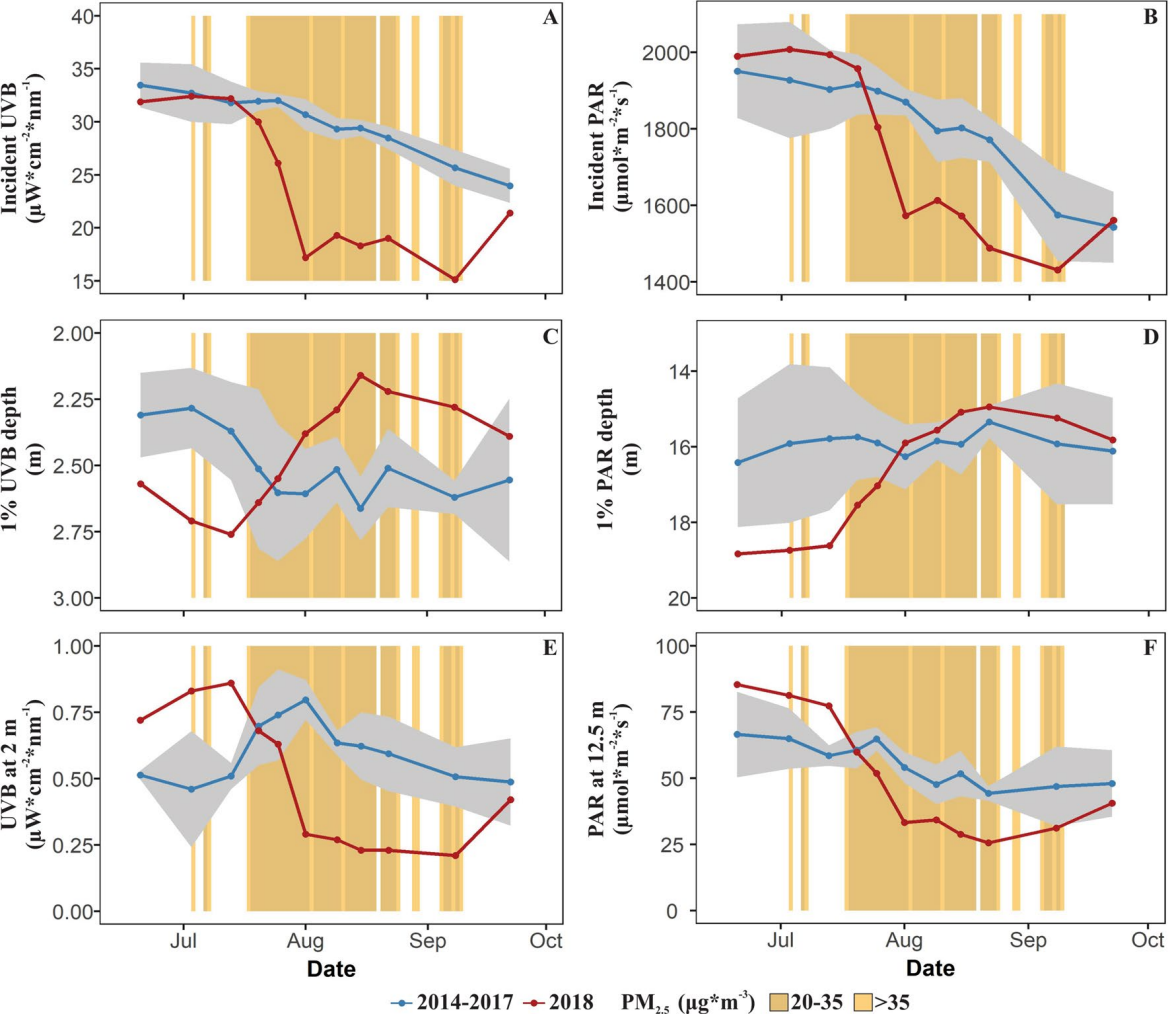
Summer seasonal pattern of incident 320 nm UV-B (**A**) and PAR (**B**) above the surface of the lake, clarity measured by the depth of 1% UV-B (**C**), and PAR (**D**), UV-B irradiance at 2 m deep (**E**), and PAR at 12.5 m deep (**F**). The 2018 year with smoke (red) is compared to the mean (± 95% confidence interval in gray) for non-smoke years from 2014 to 2017 (blue). Orange background represent days with different smoke intensities in 2018.

**Figure 4:**
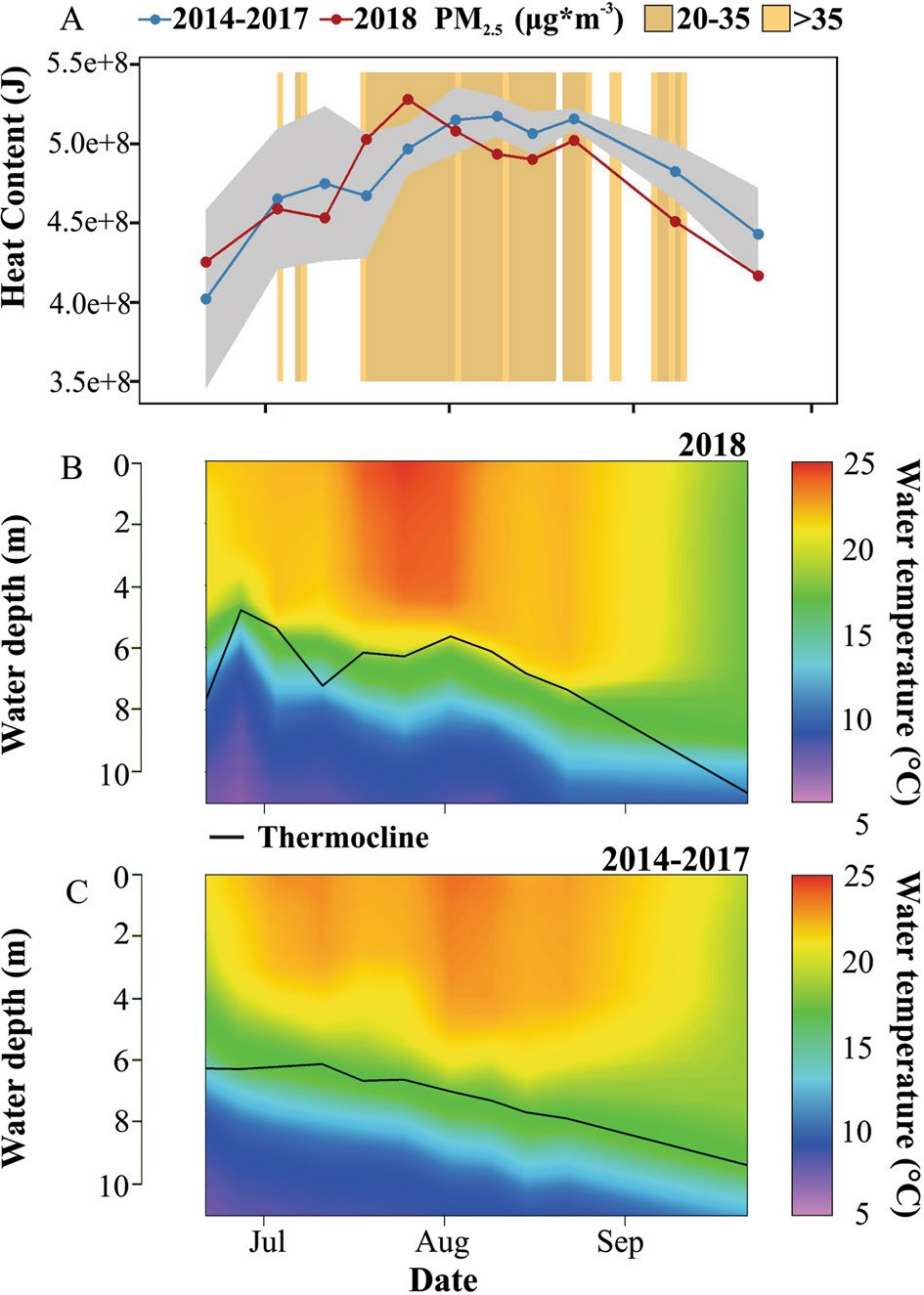
Summer seasonal pattern of the water heat content to top of the hypolimnion (10 m) of Castle lake (**A**), summer water temperature to top of the hypolimnion in 2018 (**B**), mean temperature in 2014–2017 (**C**). In panel A the values of the 2018 year with smoke (red) are compared to the mean (± 95% confidence interval in gray) for non-smoke years from 2014 to 2017 (blue). Orange background represent days with different smoke intensities in 2018.

**Figure 5:**
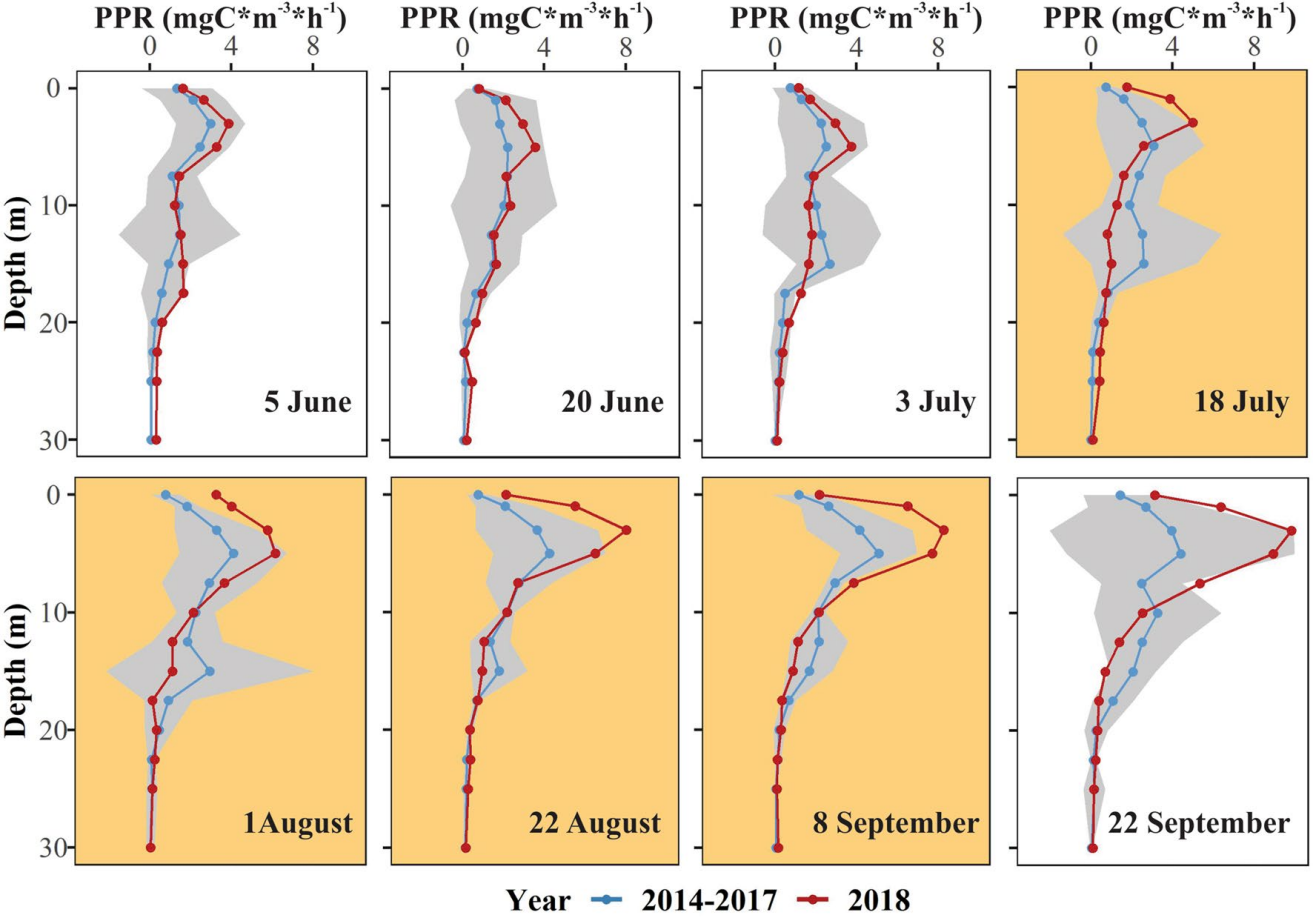
Net primary productivity (PPR) profiles comparing the smoke year of 2018 (red) to non-smoke years of 2014–2017 (blue; mean ± 95% confidence interval) in the offshore of Castle Lake. The orange background represents days with smoke in 2018.

**Figure 6:**
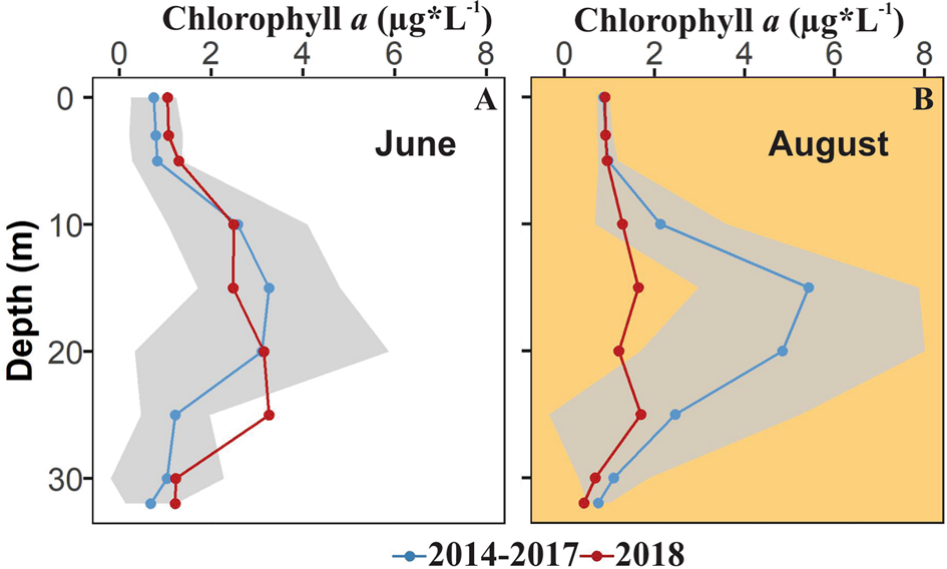
Concentration of chlorophyll *a* in 2018 and 2014–2017 (mean ± 95% confidence interval) in June (**A**) and August (**B**). The orange background represents smoke conditions on August 2018.

**Figure 7:**
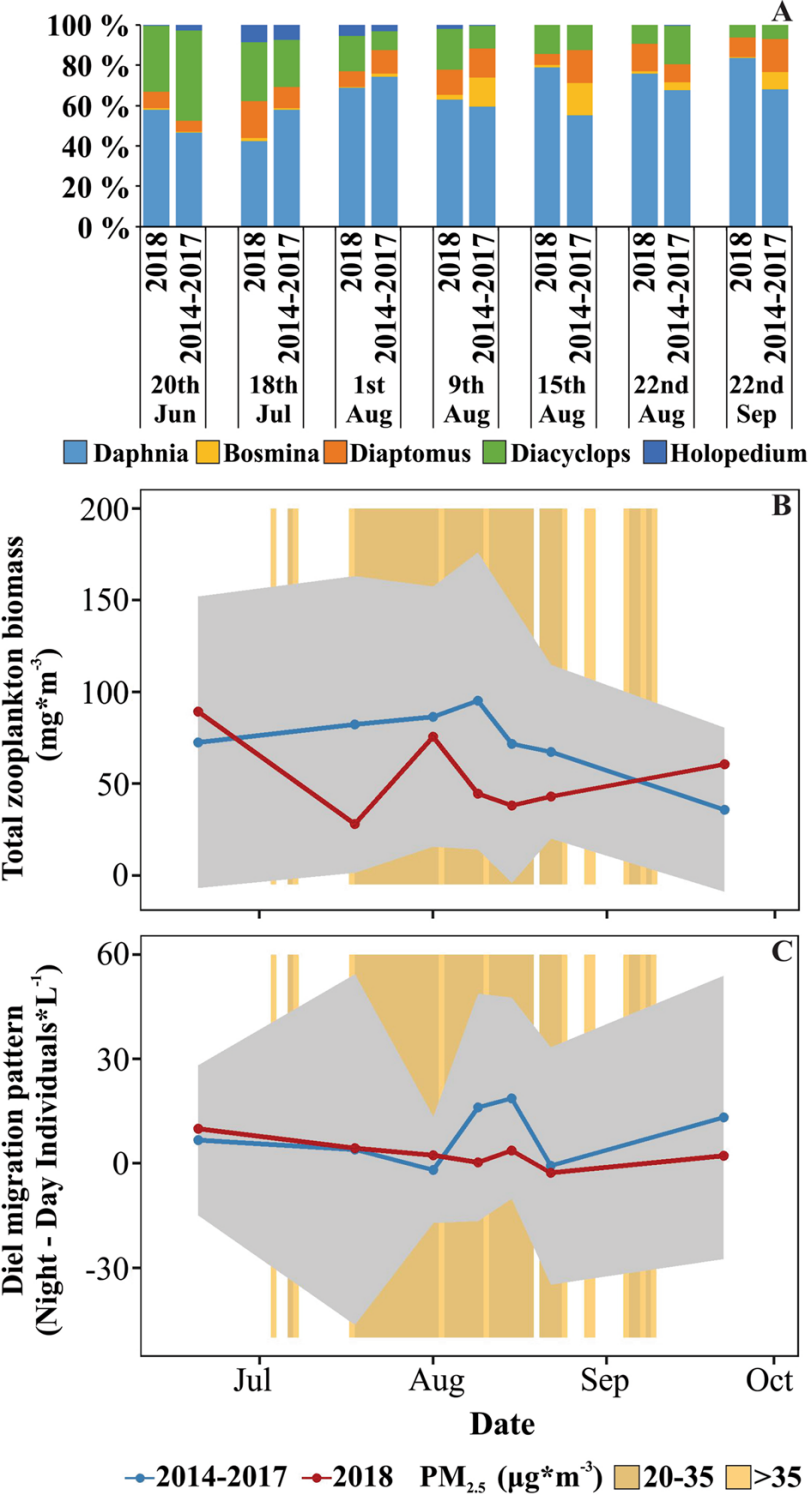
Comparison between the smoke year of 2018 (red) and 2014–2017 (blue; mean ± 95% confidence interval) of zooplankton community composition (**A**), total biomass (**B**), and migration pattern as number of individuals per liter in the night minus day at the epilimnion (**C**). Orange background represent days with different smoke intensities in 2018.

Panels on maps on Fig. 1 were done using the software ArcGIS 10.8.1^46^. Panels for Figs. 1C, 2, 3, 4, 5, 6 and 7 were done in the software R^40^. We merged the panels of the different figures using the software Inkscape^47^.

## Results

### Particulate material in the air

In 2018 we recorded shorter smoke periods on July 4 and 7–8, a longer smoke period between July 18-August 25, and other smoke periods on August 29–30, and September 5–10 (Fig. 2). Between July 18 and September 10, among all the studied years, 2018 presented a significantly (*p* < 0.001) higher number of days with smoke (87%), and also, the highest mean (64 ± 31 μg*m^−3^) and maximum (143 μg*m^−3^) value of PM_2.5_ when smoke was present (Table 1; Fig. 1C). Field notes and game camera images corroborated the PM_2.5_ data, as smoke was present on the same days when PM_2.5_ concentration increased during 2018. Additionally, the periods of increased PM_2.5_ in 2018 coincided with six major wildfires in northern California (Fig. 1D,F; Supplemental Material Sect. 1).

**Table 1:** Concentration of fine (2.5 μm of diameter) particulate matter (PM_2.5_) in the air.

Year	% of days with PM_2.5_ > 20 μg*m^-3^ (*p* value)	Mean (Standard Deviation) PM_2.5_ (μg*m^-3^)	Max PM_2.5_ (μg*m^-3^)
**2018**	**87**	**64 (31)**	**143**
2017	33 (*p* = 0.02)	48 (25)	79
2016	0	-	-
2015	40 (*p* = 0.03)	35 (12)	37
2014	40 (*p* = 0.03)	51 (21)	72

Percentage of days with smoke conditions (concentration of PM_2.5_ > 20 μg*m^−3^) between July 18 and September 10. Mean and maximum concentration of PM_2.5_ during smoke days. The highest values occurred on the smoke year and are highlighted in bold. *p* values correspond to the contrasts between 2018 and each of the years.

### Physical and chemical changes in the lake

#### Particulate carbon and nitrogen in the lake

The particulate material in the lake increased during the smoke period of 2018 compared to previous years (Fig. 2A,B). Between August 1 and September 8 of 2018 the seston particulate carbon and nitrogen in the epilimnion was in most cases above the upper limit of the 95% confidence interval of 2014–2017 (Fig. 2A,B). During that period the particulate carbon and nitrogen was on average 46 and 36%, respectively, higher than the mean of 2014–2017 (Fig. 2A,B). We also found a positive linear correlation between the concentration of PM_2.5_ and the concentration of both particulate C and N (Supplemental Material Sect. 4). The seston particulate C:N in the epilimnion in all the years was between 11 and 17 (Fig. 2C). The C:N ratio in 2018 remained similar to previous years until September when it increased (14.5) compared to the non-smoke period of 2014–2017 (11.6 ± 1.4).

#### Incident UV-B and PAR radiation

Smoke from wildfires reduced solar radiation in 2018. During the most extended smoke period (July 18–August 25) of 2018, both incident UV-B and PAR were lower and outside the 95% confidence interval of preceding years. Within the mentioned period of smoke cover, incident UV-B and PAR were 30% and 11% lower respectively in 2018 than the average of 2014–2017 (Fig. 3A,B).We observed incident UV-B and PAR decreased linearly when PM_2.5_ increased (Supplemental Material Sect. 41).

#### Water transparency measured by 1% UV-B, and 1% PAR

Metrics of water transparency indicate a decrease in the depth of light penetration in Castle Lake during the smoke periods of 2018. The light penetration was deeper than the average of previous years at the beginning of the summer in 2018, but then throughout the smoke period the depth at 1% UV-B and PAR became shallower than average (Fig. 3C,D). In 2018, during the longest smoke period, the depth of 1% UV-B decreased by 19% (0.5 m), following the reverse pattern of 2014–2017, when the depth at 1% UV-B increased during this period (Fig. 3C). The reduction in UV-B during 2018 reached values outside the 95% confidence interval of previous years. Similarly, in 2018, the depth of 1% PAR decreased by 20% (3.9 m) during the longest smoke period, while in the previous four years, depth of 1% PAR remained similar between mid-July and late August (Fig. 3D). The depth of 1% PAR during the smoke period of 2018 was substantially reduced, by 3.9 m, but the lowest depth still fell within range of previous years, likely due to the observation that water clarity in 2018 started out unusually high.

#### Light intensity at depth (2 m UV-B, and 12.5 PAR)

Smoke from wildfires reduced incident light and water transparency and therefore generated a decline in the intensity of UV-B and PAR at depth (Fig. 3E,F; Supplemental Material Sect. 4). During the most extended smoke period of 2018, PAR at the depth (12.5 m) where the deep chlorophyll *a* maximum typically develops in the lake was lower and outside the 95% confidence interval of preceding years. Within the mentioned period of smoke cover, 2 m UV-B and 12.5 m PAR were reduced in 2018 compared to 2014–2017, by 65% and 44%, respectively, (Fig. 3E,F).

#### Water temperature profiles and lake heat content

We observed decreased water column temperature and heat content during the smoke period in 2018 compared with previous years (Fig. 4A,B,C). At the beginning of the longest period of smoke cover in 2018, the epilimnetic temperature of the lake was 2.5 °C higher than in 2014–2017 (Fig. 4B,C). However, by mid-September the epilimnetic temperature decreased by 6.4 °C. The same period in 2014–2017 saw an average decrease of 3.0 °C (Fig. 4B,C). As a result of decreased epilimnetic temperatures, by August 9 (after 22 consecutive days with smoke in the lake basin) the heat content of the water decreased by 7% and was 493 * 10^6^ J, which was lower than the 95% confidence interval of the previous four years (507 * 10^6^ ± 14 * 10^6^ J), and remained low during the remainder of the summer season (Fig. 4A).

### Biological changes in the lake

#### Primary production

Primary production increased in the epilimnion and decreased in the hypolimnion during smoke cover. Overall, we observed shallow primary productivity increased linearly when PM_2.5_ increases in the basin (Supplemental Material Sect. 4). During the days with smoke in 2018, primary production in the epilimnion was 65–109% higher than the average of previous years (Fig. 5). While we observed higher epilimnetic productivity early in the season of 2018 compared to the average of 2014–2017, this pattern was enhanced during the smoke period.

In contrast, production in the hypolimnion (12.5–22.5 m), where a peak in productivity typically occurs, was 28–55% lower than the average of 2014–2017; falling within the lowest values of preceding years. Primary production in the hypolimnion decreased by 33% between early June (before the onset of the smoke) and late August in 2018. In the previous four years, primary production increased in the hypolimnion, by an average of 26% from early June to late August.

#### Phytoplankton algal biomass

During the smoke period in 2018, the chlorophyll *a* concentration in the epilimnion resembled the previous 4 years; however, the typical deep chlorophyll *a* maximum did not develop. The chlorophyll *a* concentration in June 2018 (no smoke presence) was similar to the previous 4 years (Fig. 6A), with a slight increase at 25 m. However, during August 2018 (smoke presence) the concentration of chlorophyll *a* was notably lower at 15 m (1.49 µg*L^−1^) and 20 m (1.09 µg*L^−1^) compared to the previous 4 years (15 m = 4.98 ± 1.42 µg*L^−1^; 20 m = 4.45 ± 1.84 µg*L^−1^) (Fig. 6B).

#### Nutrient limitation bioassays

The late summer nutrient limitation bioassays for phytoplankton indicate no changes in macronutrient (N and P) limitation in the smoke year of 2018 (Supplemental Material Sect. 5). The chlorophyll *a* concentration in the epilimnion was higher (*p* < 0.05) in the treatments where N and N + P were added when compared to the control for all years (Supplemental Material Sect. 5). The hierarchical logic rules^39^ indicate that the epilimnion of the lake showed N limitation during all years, regardless of smoke conditions. At the hypolimnion, nutrient additions did not produce a significant change in chlorophyll *a* in 2015–2017 or in 2018, suggesting that nutrient limitation did not inhibit development of the deep chlorophyll *a* maximum in 2018 (Supplemental Material Sect. 5).

#### Zooplankton abundance, composition, and migration

We observed a slight increase in the proportion of *Daphnia* sp. in the zooplankton community composition during the smoke period in 2018 (Fig. 7A), however neither the biomass of total zooplankton (Fig. 7B) nor the biomass of the most common genera *(Daphnia* sp., *Bosmina* sp., *Holopedium* sp., *Diacyclops* sp., *Diaptomus* sp.) differed from the previous four years (2014–2017). Both the biomass of total zooplankton and the biomass of the most common genera were lower in the smoke season of 2018, but they fell within the 95% confidence interval of 2014–2017 (Fig. 7B) indicating no statistical difference. Community composition during the smoke period was similar in 2018 to the same period in 2014–2017 (Fig. 7A; PERMANOVA, *p* = 0.22). However, before smoke covered the lake in 2018, *Daphnia* sp. represented 50 ± 7% of total zooplankton biomass, which is similar to the previous four years without smoke (52 ± 6%). During the longest period of smoke cover, *Daphnia* sp. represented 72 ± 7% of total zooplankton biomass, while between 2014 and 2017, it accounted for 64 ± 8%, suggesting an increase in *Daphnia* sp. We observed no change in the diel vertical migration pattern of total zooplankton (Fig. 7C) or any of the most common genera in the lake during the smoke period in 2018 compared to smoke-free years.

#### Fish

We captured zero trout in the benthic gill nets during the smoke period of 2018 (Supplemental Material Sect. 6), but both Rainbow and Brook trout were caught before and after the smoke period. There were other years when we captured no Rainbow trout, but the smoke period of 2018 was the first sampling with no Brook trout since 2010.

## Discussion

The smoke from regional fires (between 45 and 160 km from the lake) modified the light regime of Castle Lake basin, which altered the balance of habitat-specific production. During the 55 days in which the wildfire smoke covered the lake in 2018, the smoke attenuated the broad spectrum of solar radiation reaching the lake’s surface and reduced the exposure to UV-B radiation and PAR. As a result, the water column cooled and lost heat content while the nutrients limiting algal production remain similar to non-smoke years. Following the changes in light, we observed an increase in shallow productivity, a decrease in deep productivity, and the loss of the typical deep chlorophyll *a* maximum. The structural changes in primary production and chlorophyll *a* occurred despite the decrease in lake heat content and no change in macro nutrient limitation. Wildfire smoke had a differential influence on lake consumers. Zooplankton exhibited no statistically significant change in behavior (vertical migration), composition, and biomass, although the relative abundance of the primary grazer (*Daphnia* sp.) increased marginally. On the other hand, the dominant fishes, brook and rainbow trout, altered their behavior and disappeared from the littoral-benthic habitat during the smoke period of 2018.

In Castle Lake, the heat content and shape of the thermal profile in the lake are driven by solar heating and wind intensity^48^. We found no differences in the wind patterns between 2018 and previous years. Therefore, the decrease in incident solar radiation reaching the lake during the smoke period was likely the reason for the reduced temperature and heat content of the lake. Other studies have similarly shown that smoke plumes have the potential to cool the surface of the earth by reducing the amount of incoming solar radiation that reaches the ground^49^. Similarly, wildfire smoke decreased water temperatures of 12 rivers and streams located in the lower Klamath River Basin in northern California^50^ near our study site.

The heat content of Castle Lake typically has a strong influence on the primary productivity within the lake (literature summarized in Supplemental Material Sect. 2). From previous studies, we would have predicted that lower water temperatures and reduced heat content in 2018 would lead to less primary productivity in the lake. Instead, shallow primary production significantly increased with smoke cover. We consider three possible explanations for the productivity increase in the shallow waters during the smoky conditions: a release from photoinhibition, a stimulation from micronutrients contributed by ash deposition, and a phytoplankton community shift.

A release from photoinhibition in shallow waters in smoke conditions may be attributed to the shallower penetration of UV-B. Ultraviolet radiation causes photoinhibition of epilimnetic phytoplankton in lakes^51^. In Castle Lake, photoinhibition typically affects the top 2 m of the pelagic zone^38^ (Supplemental Material Sect. 2). Historically, Castle Lake follows a typical seasonal pattern for mesotrophic dimictic temperate lakes where 1% of UV-B becomes progressively deeper from early to late summer. This seasonal pattern is due to the photochemical degradation dissolved organic carbon (DOC), which reduces the total concentration of DOC and its UV absorbance capacity, allowing UV to penetrate to greater depths^52^. However, in 2018 we observed a stark decline in incident and underwater UV-B and PAR as the summer progressed. The particulate material in the air during the smoke cover reduced incident UV-B radiation (Fig. 2), which likely lead to lower photobleaching and photomineralization of organic carbon. Furthermore, the seston particulate organic carbon in the shallow water increased during the smoke period, which likely contributed to shallower UV-B penetration.

The increase in particulate organic carbon under the smoke conditions of 2018 was likely caused by both an increase in autochthonous and allochthonous organic matter. The range of seston C:N values (11–17) during years with and without smoke suggests that at Castle Lake the seston is composed by a mix of both algae and allochthonous terrestrial particles. C:N values for freshwater phytoplankton range from 4 to 10^53,54^, C:N values in northern California forests (mostly composed by C3 plants) range from 22 to 27^54,55^. The high primary productivity we observed during the smoke period indicates that part of the increase in particulate material could have been caused by algae growth. However, the C:N values of 2018 (13.4–14.8) indicates that regional fires may have contributed allochthonous particulate matter to the lake as might be expected from falling ash. In other aquatic ecosystems, smoke plumes have increased DOC from organic carbon deposition^56,57^. The resulting breakdown of those particles as DOC then likely altered the penetration depth of UV-B and PAR radiation^58^.

The impacts of smoke emissions on water transparency to UV-B or PAR documented in other studies varied greatly. Similar to our observations, studies from Lake Tahoe indicated that the shallow productivity increased after a reduction in PAR due to ash particles settling on the lake surface and mixing into the epilimnetic waters during the Wheeler Fire in 1985^19^. However, in Lake Tahoe in 2014 transparency to UV and PAR did not change after smoke from distant fires covered the lake^18,23^. The variable effects of smoke and ash deposition on water transparency may be related to the distance between the wildfire and the waterbody, as well as wind direction^18^. Castle Lake was located no further than 80 km downwind from the three largest wildfires in 2018, which might have favored ash deposition to the lake.

Additional nutrients contributed by ash deposition may also have stimulated production in the epilimnion^19^. After the smoke period ceased, the productivity on September 22, 2018 was higher than in 2014–2017, despite similar water transparency to UV-B. In an in-situ experiment in Lake Tahoe, the addition of dry fall-out from smoke days increased primary productivity relative to the control treatment^19^. Macronutrients did not change as a result of dry fall out, leading the researchers to conclude that the increased primary productivity may be due to trace metals in the ash deposition. Similar to this study, we found that smoke increased the particulate nitrogen and carbon in the lake but did not change the type and level of macronutrient limitation. Our bioassay experiments suggest that the shallow phytoplankton community was nitrogen limited during the smoke year of 2018 and previous years. In summary, future experiments should measure the micronutrient contributions of ash deposition and its potential to increase shallow production.

An alternative explanation to the increase in the shallow productivity may be a shift in the phytoplankton community towards more picophytoplankton, which have higher metabolic rates^59^. UV radiation causes more damage to picophytoplankton than to bigger phytoplankton^60,61^. Therefore, a reduction in UV-B may have increased the abundance of picophytoplankton. Future research on the effect of smoke conditions over the lake should focus on analyzing phytoplankton community composition.

We observed a stark decline of the magnitude of the deep chlorophyll *a* maximum and a decrease in the deep productivity during the smoke. Castle Lake’s deep-water phytoplankton is limited by light throughout the entire ice-free season^30,38^ (Supplemental Material Sect. 2). Thus, shading from particles in the upper layer controls the activity in the deeper layers. The deep-water productivity maximum often increases at the beginning to middle of the summer season in Castle Lake, with the phytoplankton living in the deeper waters to maximize chlorophyll* a* production under lower light conditions^38,62^. The smoke from the wildfires during 2018 resulted in lower intensity of PAR at the depth where the deep chlorophyll and productivity maximum usually develop. The reduction in PAR at depth was a consequence of both the decrease in incident PAR, due to particulate matter in the air, and the reduction in water transparency, likely due to the increase in shallow seston particulate matter. In a study over 100 lakes, 1% PAR was the most important factor affecting the deep chlorophyll *a* maximum development^63^, which is in line with our observed loss of the deep chlorophyll.

The absence of the deep chlorophyll *a* maximum during 2018 suggests that the phytoplankton abundance and/or community composition changed in the deeper layers of the lake. Both a decrease in available light and phytoplankton biomass resulted in a reduction of primary productivity in the deeper layers of the lake during the smoke period. The phytoplankton community in the deep chlorophyll *a* maximum at Castle Lake is composed of diatoms and large dinoflagellates^30,38^. These phytoplankton are adapted to the conditions at depth of light, temperature, and nutrients. They maintain themselves by photoautotrophic growth and not by sinking^38,64^. Given the light-limitation at these depths, any change in light might result in a phytoplankton abundance or change in the community structure. Reductions in water transparency to PAR caused by other natural hazards such as volcanic eruption modified the biomass and community composition of phytoplankton at depth^65^. Further studies should explore how wildfire smoke affects algal communities across the photic zone.

We observed no statistically significant change in zooplankton biomass, community composition or migration pattern. Zooplankton biomass during the smoke period of 2018 was lower than the average of previous years but falls within the lake’s interannual variability. We observed a slight increase in *Daphnia* sp., but overall, the zooplankton community composition was similar among the years. By mid-summer, *Daphnia* sp. typically dominate the zooplankton community in Castle Lake^30,38^ (Supplemental Material Sect. 2). In 2018, the percentage of total zooplankton biomass represented by *Daphnia* sp. increased throughout the summer more so than in previous years, although this pattern was not significant. The light conditions and/or the increase in primary productivity in the epilimnion during the smoke period of 2018 may have favored *Daphnia* sp., which adapt faster than copepods under environmental changes due to a shorter period of juvenile development^66^.

Despite the stark increase in epilimnetic primary productivity in the lake, the zooplankton biomass was lower than the mean of previous years. One hypothesis for the decrease in overall zooplankton biomass during the smoke year is related to increased grazing by trout consumers in the lake. We caught zero trout in our littoral-benthic gill nets during the smoke period in 2018; however, trout were caught in the littoral-benthic gill nets immediately after the smoke cleared from above the lake, indicating that trout temporarily moved to offshore, pelagic habitat. Trout in Castle Lake have been previously observed to be flexible in their feeding habitat, moving from near to offshore depending on water temperature and food availability^67^. In this case, one possible mechanism is that the increase in shallow productivity was followed by an increase in the rate of zooplankton production, which was high enough to draw trout away from the typically more productive littoral-benthic waters that they prefer when temperatures are suitable^67^, even though water clarity was reduced. The increase in pelagic zooplankton feeding by trout may have facilitated the low zooplankton biomass observed in this study. The ability of trout to shape zooplankton community structure has been previously noted in this lake^68^. Investigating fish behavior and feeding during smoke and non-smoke periods may enhance our understanding of the impacts of smoke on lake primary production and invertebrate biodiversity.

The diel vertical migration pattern of zooplankton did not change between the smoke period and previous years, despite the decrease in water transparency. The diel vertical migration typically occurs so that zooplankton can access the food in the surface waters at night to avoid visual predators^69^ or UV damage^70^, and then return to deep-water refuge during the day. For example, *Daphnia* sp. has receptors for both UV radiation and visible light. It is attracted to visible light but avoids UV radiation, which makes it one of the most responsive species to changes in UV and PAR^71^. Contrary to our results, previous studies showed that changes in UV penetration due to wildfire smoke, affects the vertical migration of zooplankton. The smoke from the King Fire reduced incident UV radiation by 9%, resulting in zooplankton moving up 3 m into shallower water in Lake Tahoe^23^. Changes in the behavior of zooplankton have also been detected in experiments where UV radiation was manipulated^22^. However, in Castle Lake, despite the reduction in UV-B during the smoke period of 2018 zooplankton may have continued to migrate due to the increased presence of trout in the pelagic zone. In summary, we suggest that the combined effects of decreased UV penetration and increased pelagic predation threat led to a net-zero change in the migration pattern of zooplankton.

## Conclusions

We demonstrate how atmospheric connections from fires occurring outside a basin can have cascading influences on a lake’s physical, chemical and biological dynamics. We found that smoke from wildfires reduces incident solar radiation and water transparency to UV-B and PAR, increases particulate material in the water, and increases primary productivity in the shallow waters. We also found that prolonged smoke cover significantly reduced the deep chlorophyll *a* maximum and primary productivity at depth. Traditionally these areas of production are important for supporting zooplankton. How the smoke and resulting changes in shallow and deep-water primary production influence the longer term dynamics of higher level consumers like zooplankton and fishes are less understood and warrant exploration. Investigating how animal populations alter behavior to overcome the resulting ecological changes at the base of the food web may facilitate our understanding of species persistence and recovery after wildfires.

We note that further research of connections of wildfire smoke to aquatic ecosystems is of urgent relevance given the recent increase in wildfires^9^ and expected additional increases in the coming decades^5,10^. The year 2020 was another devastating year of wildfires and smoke generation in the United States with over 10.1 million acres burned^72^ and 2.5 times the acreage burned in California compared to the year of 2018^8^. Given the increases in fires, understanding the duration, quantity and quality (e.g. particle size fraction, elemental make up and bioavailability of particles) of smoke and the influences to aquatic ecosystems could provide valuable insight into the short and long term ecological changes which may manifest in a lake, river, or estuary. The concentration of particulate matter in the air during smoke periods correlated with specific environmental variables (e.g. incident light at the surface and light wavelengths within the lake at depth) but there were no direct relationships with other parameters like heat content and algal biomass measured as chlorophyll a (see Supplemental Sect. 4). This suggests that there may be some direct responses that can be attributed to changes in smoke quantity, however, there are likely slower, delayed responses which may occur depending on the extent and duration of the smoke. In short, direct and indirect along with additive responses may occur depending on the duration and intensity of smoke above an ecosystem. Setting up future work to quantify the relationship between smoke quality and quantity from specific fires within a landscape will be important if we are to quantify the time dependent responses to an ecosystem. Understanding within season responses to specific fires burning within a region along with repeated effects of smoke occurring across years is a bright area of investigation given the increased combined drought-wildfire events that are occurring in recent years. Finally, while our study investigates the response of a single lake ecosystem to regional wildfire smoke, the fact that smoke can impact the air at the continental level as evidenced by the 2020 wildfire season^72^ suggests that a transdisciplinary approach is needed to quantify how much and what type of smoke is generated from a fire(s), determine where smoke will move across regional to continental airsheds, and understand whether or not the ecosystems “receiving” the smoke are resilient to the perturbations due to their initial ecological and watershed characteristics.

## Supplementary Information

Supplementary Information.

## Data Availability

Data generated or analyzed during this study are included in the Supplementary Information Sect. 7. Yreka PM2.5 Data is available at https://www.epa.gov/outdoor-air-quality-data/download-daily-data.
