# Supplementary material for: Smoke from regional wildfires alters lake ecology
Source: Sci Rep. 2021 May 25;11:10922. doi: 10.1038/s41598-021-89926-6 (PMC8149697; doi:10.1038/s41598-021-89926-6)
Supplement: Supplementary file 1 — Supplementary Information. [file 41598_2021_89926_MOESM1_ESM.pdf]

Smoke from Regional Wildfires Alters Lake Ecology

Facundo Scordo, Sudeep Chandra, Erin Suenaga, Suzanne J. Kelson, Joshua Culpepper, Lucia Scaff, Flavia Tromboni, Timothy J Caldwell, Carina Seitz, Juan E. Fiorenza, Craig E. Williamson, Steven Sadro, Kevin C. Rose, Simon R. Poulson

Supplemental material

Section 1

Table Section 1. Fires near Castle Lake during 2018 ice-free season.
Name of fire, area burned, dates, and the distance from Castle Lake of the six largest wildfires that occurred in California during the spring and summer 2018, within a radius of 200 km from the lake (data source[1,2]).

Name of Fire	Area burned (km^2)	Dates of fire in 2018	Distance from Castle Lake (km)
Carr	926	23 Jul to 30 Aug	80
Delta	255	05 Sep to 07 Oct	45
Hirz	186	09 Aug to 12 Sep	50
Stone	160	15 to 29 Aug	150
Naztche	154	15 Jul to 30 Oct	160
Klamathon	153	05 Jul to 16 Jul	100

Section 2

What do we know about Castle Lake in years without smoke conditions?

Published research about Castle Lake's ecology in the last 59 years provides a detailed understanding of the drivers of pelagic productivity and other biological variables in this waterbody, which we summarized in our conceptual model in the Table Section 4. The main factors affecting the inter-annual productivity in Castle Lake are ice-out date, snow water equivalent (SWE) of the snow pack, water temperature and the resulting heat content, and primary productivity from the previous year [3–5]. A larger snow pack increases the water flux into the lake, which flushes the phytoplankton and the nutrients out of the lake. Late ice out date, results in lower heat content during the summer and shortens the time the phytoplankton community has to recover, resulting in lower productivity [6]. A deep chlorophyll and productivity maximum develop between 12.5 and 20 m readily after ice breakup. In general, more than half of the total chlorophyll in the lake is located below 12.5 m [7,8]. Deep-water productivity within the depth range of the deep chlorophyll *a* maximum is controlled by shading from epilimnetic production by mid-summer [4,8,9]. Zooplankton (herbivorous grazers like daphnids) play an important role in regenerating nutrients (N) in the epilimnion or exerting top-down controls on algae during late summer [10–12]. By the end of the summer, light decline becomes a more important limitation than nutrient availability to primary productivity [4,8]. Therefore, by the end of the summer, zooplankton predation on algae overcomes their nutrient contribution to epilimnetic waters, which negatively affects primary productivity [4,13,14]. Other factors, such as trout feeding behavior can affect the zooplankton community and influence the primary productivity in the lake [4,15]. The trout can feed on late-summer *Daphnia* sp. populations, which in turn graze on the mixed-layer

algal community [4,15].

Table Section 2 Description of the main drivers of Castle Lake pelagic primary productivity based on publications to date.

**Inter-annual primary productivity depends on**			
**Snow ice out date**<br>-High SWE, increased the flush of the phytoplankton and the nutrients out of the lake	**Ice out date**<br>-Late ice out reduce the time the phytoplankton has to recovered from the flush, and the supply of nutrients via spring mixing	**Water temperature**<br>-Increase metabolic rates<br>-Accelerate nutrients regeneration rate	**PPR from previous years**<br>-Higher PPR and consequently anoxic layer previous years increase lead to an increase in the release of nutrients to the water column available for the subsequent year

**Intra-annual primary productivity depends on**			
**Lake depth**	**Early summer**	**Mid-summer**	**Late summer**
**Surface water (0-2 m)**	**Light inhibition**		
**Bottom of mix layer (3-10m)**<br>**PPR controlled by physical, chemical, and biological drivers**	**Low PPR, phytoplankton and zooplankton biomass**<br>-Nutrients phytoplankton, and zooplankton community flushed out of the lake. PPR affected by light inhibition, nutrient limitation, but not by zooplankton.<br>-Zooplankton community (dominate by copepods which presents low DVM) also affected by trouts which remain in the upper water until mid-summer	**High PPR, phytoplankton and zooplankton biomass**<br>-Phytoplankton and zooplankton community recovered from flush out<br>**High zooplankton biomass with strong DVM (*Daphnia* sp.) positive effect on phytoplankton (regeneration of Nh4+)**<br>**Low trout population at these depth**	**Low PPR and phytoplankton biomass**<br>-Phytoplankton community light limited<br>High zooplankton biomass with strong DVM **(*Daphnia* sp.) negative effect on phytoplankton (predation), which can be controlled by the zooplanktivorous community composition and trouts feeding position**
	**Phytoplankton community composition highly variable, dominated mostly by blue-green and green algae**		
**Deep chlorophyll *a* maximum (12.5- 20 m)**<br>**PPR controlled by physical drivers**	**High PPR and phytoplankton biomass (deep chlorophyll *a* maximum)**<br>-Phytoplankton community is less flushed at this depth<br>- Nutrient storage is higher	**High phytoplankton biomass (deep chlorophyll *a* maximum), decrease in PPR**<br>- Shading from epilimnetic algae community (light limitation)	**Low phytoplankton biomass, low PPR starting at 10 m**
	**Phytoplankton community composition dominated by diatoms and dinoflagellates (**adapted to the conditions of light, temperature, and nutrients, and maintain themselves by photoautotrophic growth and not by sinking)		
**Deep-water (25-30 m)**	**Light limitation due to shading**		

Section 3

Table Section 3. Description of variables used in this study.
Variables presented for this study collected as part of the Castle Lake Environmental Research and Education Program. Units, temporal and depth resolution of the variable are presented. Epi = epilimnion (surface layers of the pelagic zone), Hypo = hypolimnion (bottom waters of the lake).

**Variable**	**Units**	**Temporal resolution**	**Depth resolution**
$PM_{2.5}$ in the air	µg $*m^{-3}$	Daily to weekly	
Particulate C and N in water	µg$*L^{-1}$	bi-weekly	Epi (0 , 3, 5 m)
Surface and subsurface PAR	µmol$*m^{-2}*s^{-1}$	weekly	~ 0.1 m
Surface and subsurface UV-B	mW$*cm^{-2}*nm^{-1}$	weekly	~ 0.1 m
Water temperature profiles	°C	weekly	~ 0.1 m
Net primary productivity	mg C $*m^{-3}*h^{-1}$	bi-weekly	0, 1, 3, 5, 7.5, 10, 12.5, 15, 17.5, 20, 22.5, 25, 30 m
Chlorophyll *a*	µg$*L^{-1}$	bi-weekly	0, 3, 5, 10, 15, 20, 25, 30, 32 m
Zooplankton biomass	µg$*L^{-1}$	weekly	Epi (0, 3, 5 m)
Zooplankton migration	individuals$*L^{-1}$ (night-day)	weekly	Epi (0, 3, 5 m)
Fish catch	Catch per unit effort (individuals$*h^{-1}$)	monthly	Benthic shallow gill nets (0-2 m)<br>Benthic deep gill nets (3-5 m)
Bioassay *(*chlorophyll *a* as response variable)	µg$*L^{-1}$	August	Epi (0, 3, 5 m)<br>Hypo (~ 15 m)

Section 4

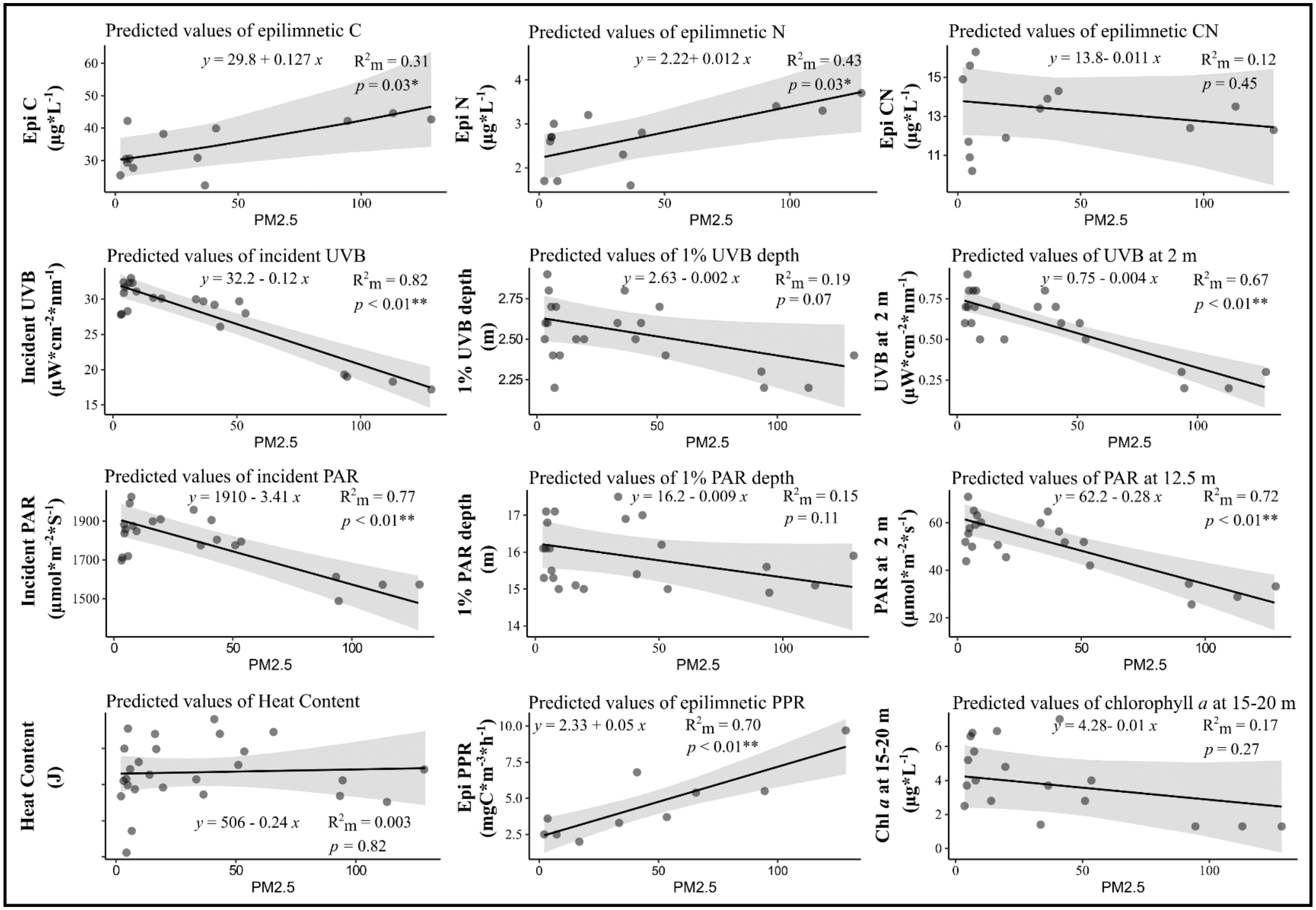

Predicted values of epilimnetic C
$y = 29.8 + 0.127x$
$R^2_m = 0.31$
$p = 0.03$*
Epi C ($\mu g*L^{-1}$)
PM2.5
Predicted values of epilimnetic N
$y = 2.22 + 0.012x$
$R^2_m = 0.43$
$p = 0.03$*
Epi N ($\mu g*L^{-1}$)
PM2.5
Predicted values of epilimnetic CN
$y = 13.8 - 0.011x$
$R^2_m = 0.12$
$p = 0.45$
Epi CN ($\mu g*L^{-1}$)
PM2.5
Predicted values of incident UVB
$y = 32.2 - 0.12x$
$R^2_m = 0.82$
$p < 0.01$**
Incident UVB ($\mu W*cm^{-2}*nm^{-1}$)
PM2.5
Predicted values of 1% UVB depth
$y = 2.63 - 0.002x$
$R^2_m = 0.19$
$p = 0.07$
1% UVB depth (m)
PM2.5
Predicted values of UVB at 2 m
$y = 0.75 - 0.004x$
$R^2_m = 0.67$
$p < 0.01$**
UVB at 2 m ($\mu W*cm^{-2}*nm^{-1}$)
PM2.5
Predicted values of incident PAR
$y = 1910 - 3.41x$
$R^2_m = 0.77$
$p < 0.01$**
Incident PAR ($\mu mol*m^{-2}*S^{-1}$)
PM2.5
Predicted values of 1% PAR depth
$y = 16.2 - 0.009x$
$R^2_m = 0.15$
$p = 0.11$
1% PAR depth (m)
PM2.5
Predicted values of PAR at 12.5 m
$y = 62.2 - 0.28x$
$R^2_m = 0.72$
$p < 0.01$**
PAR at 2 m ($\mu mol*m^{-2}*s^{-1}$)
PM2.5
Predicted values of Heat Content
$y = 506 - 0.24x$
$R^2_m = 0.003$
$p = 0.82$
Heat Content (J)
PM2.5
Predicted values of epilimnetic PPR
$y = 2.33 + 0.05x$
$R^2_m = 0.70$
$p < 0.01$**
Epi PPR ($mgC*m^{-3}*h^{-1}$)
PM2.5
Predicted values of chlorophyll *a* at 15-20 m
$y = 4.28 - 0.01x$
$R^2_m = 0.17$
$p = 0.27$
Chl *a* at 15-20 m ($\mu g*L^{-1}$)
PM2.5

Figure Section 4. Correlations between $PM_{2.5}$ (as a surrogate of smoke) concentration and limnological variables using linear mixed-effects models (LMM). Panels presents the slopes of the models and it associated *p*-values (values lower than 0.05 are significant), and the marginal R^2. We observed positive linear correlations between $PM_{2.5}$ and particulate carbon (Epi C), particulate nitrogen (Epi N), and primary productivity (Epi PPR) in the shallow water of the lake. We observed a negative linear correlation between $PM_{2.5}$ and the incident and under water 320 nm UV-B and PAR. Other variables such as the concentration of chlorophyll *a* in the deep waters of the lake or the heat content of the lake did not present a linear correlation with the concentration of $PM_{2.5}$; there are likely more complex dynamics that lead to the lack of direct correlation between specific $PM_{2.5}$ and these variables along with time lag responses that are not detected through one to one linear analysis. For example, we do find a strong correlation between chlorophyll *a* at the deep water of the lake and the previous 30 days average of $PM_{2.5}$ ($\beta 1$ = -0.05, p =0.02, R^2_m.= 0.3)

Section 5

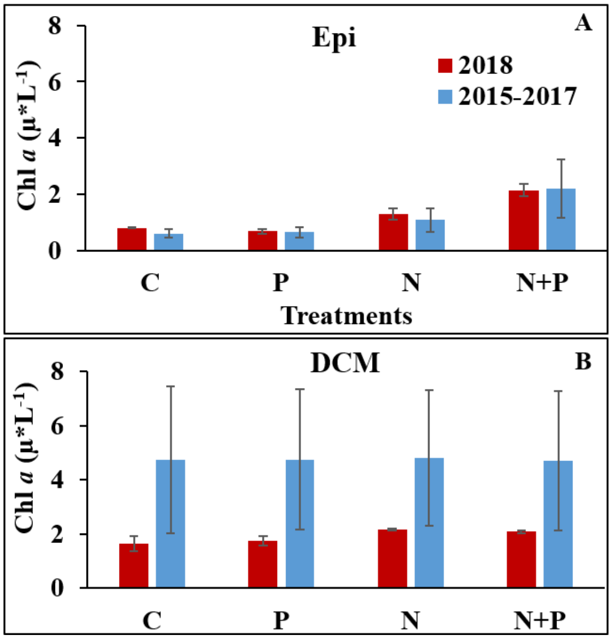

Figure Section 5. Comparison of the results from the bioassays run at the Castle Lake epilimnion (Epi; A) and DCM (B) in August between the smoke year of 2018 (red) and 2010-2017 (blue). When comparing 2018 to 2015-2017, in both the epilimnion and DCM, the effects over the concentration of chl *a* produced by the different treatments presented no significant differences ($p \geq 0.08$).

Section 6

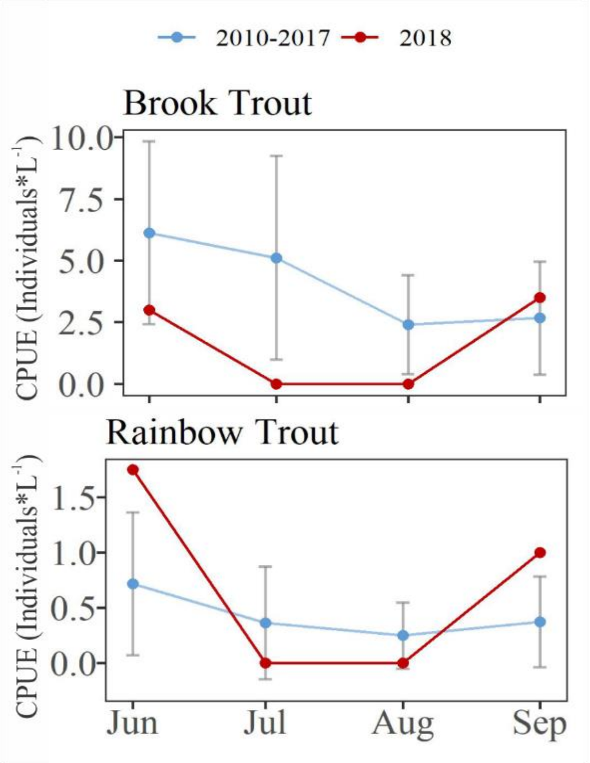

Figure Section 6. Comparison between the smoke year of 2018 (red) and 2010-2017 (blue, mean ± 95% confidence interval) of fish catch per unit effort (CPUE; individuals*h^{-1}) from the nearshore-littoral zone during the summer seasons. No trout were captured during the smoke season of 2018. However, fish were caught before and after the period of smoke.

References Supplemental materials

1. Fire, C. https://www.fire.ca.gov/incidents/2018/. *https://www.fire.ca.gov/incidents/2018/* (2020). Available at: https://www.fire.ca.gov/incidents/2018/.
2. MTBS. https://www.mtbs.gov/. *MTBS* (2020). Available at: https://www.mtbs.gov/.
3. Goldman, C. R., Jassby, A. & Powell, T. Interannual fluctuations in primary production: Meteorological forcing at two subalpine lakes. *Limnol. Oceanogr.* **34**, 310–323 (1989).
4. Jassby, A. D., Powell, T. M. & Goldman, C. R. Interannual Fluctuations in Primary Production: Direct Physical Effects and the Trophic Cascade at Castle Lake, California. *Limnol. Oceanogr.* **35**, 1021–1038 (1990).
5. Park, S., Brett, M. T., Müller-Solger, A. & Goldman, C. R. Climatic forcing and primary productivity in a subalpine lake: Interannual variability as a natural experiment. *Limnol. Oceanogr.* **49**, 614–619 (2004).
6. Strub, P. T., Powell, T. & Goldman, C. R. Climatic forcing: Effects of El Niño on a small, temperate lake. *Science (80-.).* **227**, 55–57 (1985).
7. Priscu, J. C. & Goldman, C. R. The effect of temperature on photosynthetic and respiratory electron transport system activity in the shallow and deep-living phytoplankton of a subalpine lake. *Freshw. Biol.* **14**, 143–155 (1984).
8. Huovinen, P. S., Brett, M. T. & Goldman, C. R. Temporal and vertical dynamics of phytoplankton net growth in Castle Lake, California. *J. Plankton Res.* **21**, 373–385 (1999).
9. Priscu, J. C. & Goldman, C. R. Seasonal dynamics of the deep-chlorophyll maximum in Castle Lake, California. *Can. J. Fish. Aquat. Sci.* **40**, 208–214 (1983).
10. Axler, R. P., Redfield, G. W. & Goldman, C. R. The Importance of Regenerated Nitrogen to Phytoplankton Productivity to Phytoplankton Productivity in a Subalpine Lake. *Ecology* **62**, 345–354 (1981).
11. Axler, R. P., Gersberg, R. M. & Goldman, C. R. Inorganic nitrogen assimilation in a subalpine lake. *Limnol. Oceanogr.* **27**, 53–65 (1982).
12. Zehr, J. P., Axler, R. P. & Goldman, C. R. Heterotrophic mineralization of amino acid nitrogen in subalpine Castle Lake, California. *Mar. Chem.* **16**, 343–350 (1985).
13. Redfield, G. W. & Goldman, C. R. Diel vertical migration and dynamics of zooplankton biomass in the epilimnion of Castle Lake, California. *Verhandlungen des Int. Verein Limnol.* **20**, 381–387 (1978).
14. Elser, J. J. & Goldman, C. R. Zooplankton effects on phytoplankton contrasting trophic status. **36**, 64–90 (1988).
15. Elser, J. J., Luecke, C., Brett, M. T. & Goldman, C. R. Effects of Food Web Compensation After Manipulation of Rainbow Trout in an Oligotrophic Lake. *Ecology* **76**, 52–69 (1995).

Section 7 Data

Particulate Carbon (μg*m^{-3})

Datetime	2018	2017	2016	2015	2014
6/5/2018	32.9		46.5	26.8	34.2
6/20/2018	29.9	28.6	31.0	26.8	27.6
7/3/2018	22.8	17.7		25.3	27.2
7/18/2018	30.8	27.7	30.5	31.1	30.9
8/1/2018	42.7	39.9	29.3	25.4	42.2
8/15/2018	44.6	38.2	30.6	22.3	34.5
8/22/2018	42.2	38.2	30.6	29.3	
9/8/2018	56.3		29.7	19.1	41.8
9/22/2018	40.3	57.2	20.3	22.8	35.0

Particulate Nitrogen (μg*m^{-3})

Datetime	2018	2017	2016	2015	2014
6/5/2018	2.5		3.6	1.6	2.7
6/20/2018	2.2	1.9	2.1	1.6	2.6
7/3/2018	1.7	1.2		1.8	2.6
7/18/2018	2.3	1.7	2.6	2.3	2.8
8/1/2018	3.7	2.8	2.7	1.7	2.7
8/15/2018	3.3	3.2	3.0	1.6	2.8
8/22/2018	3.4	3.2	3.0	2.3	2.8
9/8/2018	3.8		2.4	1.5	3.8
9/22/2018	2.9	4.1	1.8	1.9	3.0

C:N ratio

Datetime	2018	2017	2016	2015	2014
6/5/2018	13.2		12.9	16.8	12.7
6/20/2018	13.6	15.1	14.8	16.8	10.6
7/3/2018	13.4	14.8		14.1	10.5
7/18/2018	13.4	16.3	11.7	13.5	11.0
8/1/2018	12.3	14.3	10.9	14.9	15.6
8/15/2018	13.5	11.9	10.2	13.9	12.3
8/22/2018	12.4	11.9	10.2	12.7	
9/8/2018	14.5	13.9	12.4	12.7	11.0
9/22/2018	13.9	14.0	11.3	12.0	11.7

Surface UVB (μW*cm^{-2}*nm^{-1})

Datetime	2018	2017	2016	2015	2014
6/20/2018	31.9	35.5	33.1	31.8	
7/3/2018	32.4	35.2	32.6	30.4	
7/13/2018	32.2	33.5	31.9	29.9	
7/20/2018	30.0	33.0	30.9	31.5	32.4
7/25/2018	26.1	32.3	32.3	31.1	32.4
8/1/2018	17.2	29.2	31.7	31.1	
8/9/2018	19.3	30.2	27.8	29.7	29.6
8/15/2018	18.3	30.1	28.3	29.7	29.5
8/22/2018	19.0	28.0	27.9		29.6
9/8/2018	15.1		25.7	24.1	27.1
9/22/2018	21.4	24.1	22.9	22.7	26.3

1% UVB (m)

Datetime	2018	2017	2016	2015	2014
6/20/2018	2.57	2.16	2.33	2.44	
7/3/2018	2.71	2.13	2.38	2.34	
7/13/2018	2.76	2.23	2.55	2.33	
7/20/2018	2.64	2.24	2.55	2.33	2.93
7/25/2018	2.55	2.37	2.70	2.41	2.93
8/1/2018	2.38	2.46	2.76		
8/9/2018	2.29	2.48	2.50	2.69	2.60
8/15/2018	2.16	2.53	2.71	2.81	2.60
8/22/2018	2.22	2.36	2.57		2.60
9/8/2018	2.28		2.61	2.68	2.57
9/22/2018	2.39	2.10	2.82	2.68	2.62

UVB at 2 m (μW*cm^{-2}*nm^{-1})

Datetime	2018	2017	2016	2015	2014
6/20/2018	0.72	0.51	0.53	0.5	
7/3/2018	0.83	0.25	0.63	0.5	
7/13/2018	0.86	0.46	0.54	0.53	
7/20/2018	0.68	0.65	0.72	0.53	0.89
7/25/2018	0.63	0.76	0.82	0.49	0.89
8/1/2018	0.29	0.72	0.84		
8/9/2018	0.27	0.69	0.6	0.59	0.66
8/15/2018	0.23	0.45	0.62	0.76	0.66
8/22/2018	0.23	0.45	0.67		0.66
9/8/2018	0.21		0.46	0.44	0.62
9/22/2018	0.42	0.27	0.61	0.44	0.63

Surface PAR (μmol*m^{-2}*s^{-1})

Datetime	2018	2017	2016	2015	2014
6/20/2018	1989.6	2073.4	1913.5	1866.5	
7/3/2018	2007.6	2073.5	1900.3	1808.7	
7/13/2018	1993.8	2007.5	1869.8	1833.4	
7/20/2018	1958.0	2025.1	1836.6	1920.1	1882.4
7/25/2018	1804.4	1991.3	1874.7	1848.0	1882.4
8/1/2018	1573.0	1905.3	1857.1		
8/9/2018	1612.6	1899.2	1697.2	1774.9	1807.5
8/15/2018	1572.1	1908.9	1718.6	1775.0	1807.5
8/22/2018	1488.3	1793.9	1712.4		1807.6
9/8/2018	1431.0		1596.4	1459.0	1667.8
9/22/2018	1561.1	1612.8	1464.1	1458.7	1635.8

1% PAR (m)

Datetime	2018	2017	2016	2015	2014
6/20/2018	18.83	15.99	18.09	15.17	
7/3/2018	18.74	15.2	18.02	14.51	
7/13/2018	18.62	15.27	17.66	14.43	
7/20/2018	17.54	15.25	17.14	14.43	16.13
7/25/2018	17.03	15.46	17.06	14.95	16.13
8/1/2018	15.90	15.39	16.77		
8/9/2018	15.56	15.1	16.12	16.19	15.73
8/15/2018	15.08	14.96	16.1	16.94	15.73
8/22/2018	14.94	14.96	15.33		15.73
9/8/2018	15.24		15.44	17.52	14.81
9/22/2018	15.82	15.06	17.17	17.52	14.70

PAR at 12.5 m (μmol*m^{-2}*s^{-1})

Datetime	2018	2017	2016	2015	2014
6/20/2018	85.4	51.6	80.0	67.9	
7/3/2018	81.3	56.6	76.2	62.1	
7/13/2018	77.3	56.3	62.5	56.9	
7/20/2018	59.9	59.1	55.4	56.9	70.9
7/25/2018	51.8	65.1	63.1	60.2	70.9
8/1/2018	33.2	56.3	57.7		48.2
8/9/2018	34.3	50.6	51.9	51.9	36.2
8/15/2018	28.8	45.5	49.9	64.7	46.9
8/22/2018	25.6	42.0	43.8		46.9
9/8/2018	31.1		38.3	62.3	40.2
9/22/2018	40.5	32.3	53.2	62.3	44.2

Heat content (Joules)

Datetime	IntE2018	IntE2017	IntE2016	IntE2015	IntE2014
6/21/2018	425595483	331703684	381189041	461557236	434821742
7/3/2018	458983462	404468990	463990355	513057080	479651930
7/11/2018	453454276	425410856	439002950	518007652	517516480
7/18/2018	502895834	412548194	462332955	491669314	503187215
7/25/2018	528031477	474339185	497392349	512462658	503187215
8/2/2018	508321021	536130176	499726176	493526120	530931585
8/9/2018	493717006	527955308	502060004	510838035	529053888
8/15/2018	490393959	498368627	508506706	494473172	525574434
8/22/2018	502335823	518287073	519877146	505459342	519631557
9/8/2018	450946971	500832967	464569717	469524012	495415137
9/22/2018	416988101	483378861	421653954	419576863	448389524

Water temperature 2018 (°C)

dateTime	6/21	6/27	7/3	7/11	7/18	7/25	8/2	8/9	8/15	8/22	9/22
wtr_0.0	20.0	20.3	20.8	21.0	23.0	23.8	23.0	21.7	20.6	20.9	16.3
wtr_0.5	19.8	20.3	20.7	20.7	22.9	23.6	23.0	21.4	20.6	20.9	16.2
wtr_1.0	19.2	20.2	20.7	20.6	22.6	23.4	22.9	21.2	20.5	20.8	16.1
wtr_1.5	19.0	20.2	20.6	20.5	22.4	23.2	22.9	21.2	20.5	20.9	16.0
wtr_2	19.0	20.1	20.5	20.4	22.3	23.1	22.8	21.2	20.5	20.8	16.0
wtr_2.5	18.9	20.0	20.5	20.3	22.2	23.0	22.8	21.2	20.5	20.7	16.0
wtr_3	18.8	19.7	20.5	20.2	22.1	23.0	22.8	21.2	20.4	20.7	16.0
wtr_3.5	18.8	18.7	20.4	20.1	22.1	22.9	22.7	21.1	20.4	20.7	16.0
wtr_4.0	18.8	17.6	20.4	20.0	21.8	22.8	22.6	21.0	20.4	20.7	16.0
wtr_4.5	18.4	16.6	20.4	19.7	21.2	22.3	22.5	21.0	20.4	20.7	16.0
wtr_5.0	17.6	13.4	19.6	18.7	20.6	21.2	21.4	21.0	20.4	20.7	16.0
wtr_5.5	15.8	11.3	16.8	17.1	19.1	19.2	19.3	20.8	20.3	20.7	15.9
wtr_6.0	13.7	10.3	14.4	14.5	17.5	18.3	16.3	19.0	20.3	20.6	16.0
wtr_6.5	11.9	9.3	12.9	12.8	15.5	16.1	14.6	16.8	19.8	20.4	16.0
wtr_7.0	10.7	8.5	11.8	11.9	13.8	14.6	13.2	15.2	16.6	19.4	15.9
wtr_7.5	10.1	7.8	10.8	10.7	12.4	13.2	12.0	13.5	14.3	17.1	15.9
wtr_8.0	9.3	7.3	10.1	9.6	11.0	11.8	10.8	11.6	12.8	15.1	15.8
wtr_8.5	8.7	7.0	9.5	8.9	10.2	10.9	10.2	10.3	11.6	13.5	15.5
wtr_9.0	8.2	6.7	8.8	8.4	9.4	10.1	9.3	9.5	10.6	12.4	14.1
wtr_9.5	7.6	6.5	8.3	8.0	8.8	9.4	8.6	8.8	9.7	11.5	12.2
wtr_10.0	7.1	6.3	7.8	7.6	8.3	8.8	8.1	8.2	9.1	10.7	11.0

Water temperature average 2014-2017 (°C)

dateTime	6/21	6/27	7/3	7/11	7/18	7/25	8/2	8/9	8/15	8/22	9/22
wtr_0.0	19.1	20.3	21.9	22.0	21.2	21.3	22.7	22.2	21.1	21.7	17.5
wtr_0.5	19.0	20.2	21.7	22.0	21.1	21.2	22.5	22.2	21.5	21.7	17.4
wtr_1.0	18.6	20.0	21.3	21.9	21.0	21.2	22.4	22.1	21.4	21.6	17.2
wtr_1.5	18.1	19.8	21.0	21.8	20.9	21.1	22.3	22.0	21.3	21.5	17.1
wtr_2	17.8	19.6	20.9	21.8	20.8	21.0	22.2	21.9	21.2	21.5	17.1
wtr_2.5	17.6	19.5	20.8	21.7	20.7	20.9	22.1	21.8	21.2	21.4	17.0
wtr_3	17.2	19.3	20.6	21.1	20.0	20.3	22.0	21.8	21.2	21.4	17.0
wtr_3.5	16.7	18.6	19.8	20.5	19.5	19.5	21.9	21.7	21.2	21.3	17.0
wtr_4.0	16.1	17.9	18.8	19.5	18.8	19.0	21.7	21.7	21.0	21.1	17.0
wtr_4.5	15.3	17.2	18.0	18.7	18.3	18.7	21.2	21.4	20.7	20.4	17.0
wtr_5.0	14.4	16.3	17.0	18.0	17.8	18.4	20.5	20.4	20.1	19.8	16.9
wtr_5.5	13.4	15.3	15.8	16.9	17.1	17.9	19.6	19.2	19.5	19.1	16.9
wtr_6.0	12.6	14.3	14.8	15.7	16.3	16.9	18.5	18.1	19.0	18.4	16.9
wtr_6.5	11.6	13.0	13.5	14.3	15.1	15.7	17.5	17.1	18.2	17.5	16.8
wtr_7.0	10.5	11.7	12.3	13.1	13.6	14.2	16.1	16.0	17.1	16.7	16.8
wtr_7.5	9.7	10.7	11.4	11.9	12.4	12.8	14.5	14.7	16.0	15.7	16.7
wtr_8.0	8.8	10.0	10.5	10.9	11.3	11.6	13.1	13.4	14.6	14.4	16.4
wtr_8.5	8.2	9.4	9.7	10.1	10.4	10.7	11.9	12.2	13.4	13.1	15.9
wtr_9.0	7.7	8.7	9.0	9.5	9.5	9.9	10.9	11.2	12.3	12.0	15.2
wtr_9.5	7.2	8.2	8.4	8.9	8.9	9.2	10.1	10.4	11.4	11.1	14.0
wtr_10.0	6.8	7.6	7.9	8.3	8.4	8.5	9.4	9.6	10.5	10.2	12.8

August chlorophyll *a* ($\mu*L^{-1}$)

Depth	chla2014	chla2015	chla2016	chla2017	chla2018
0	0.71	0.69	0.84	0.83	0.79
3	0.75	0.71	0.85	0.94	0.81
5	0.75	0.78	0.89	1.03	0.84
10	1.43	1.60	3.21	1.53	1.16
15	4.01	4.19	4.64	7.08	1.49
20	6.34	1.93	4.89	4.62	1.09
25	4.59	1.09	2.07	1.23	1.54
30	1.63	0.72	0.63	0.93	0.60
32	0.57	0.68	0.56	0.87	0.37

June chlorophyll *a* (µ*L^{-1})

Depth	chla2014	chla2015	chla2016	chla2017	chla2018
0	0.56	1.17	0.79	0.49	1.05
3	0.53	1.25	0.96	0.47	1.08
5	0.60	1.28	0.88	0.53	1.29
10	1.38	3.20	3.48	2.27	2.50
15	3.84	4.04	3.33	1.87	2.48
20	4.11	4.87	2.49	0.96	3.15
25	1.01	1.75	1.45	0.68	3.26
30	0.45	2.08	1.20	0.46	1.24
32	0.43	0.95	1.01	0.35	1.23

Zooplankton biomass (mg*m^{-3})

Datetime	Zoo2018	Zoo2017	Zoo2016	Zoo2015	Zoo2014
6/20/2018	89.4	41.3	142.2	32.3	75.0
7/18/2018	28.1	70.0	150.2	82.0	27.7
8/1/2018	75.7	116.6	122.6	82.0	25.3
8/9/2018	44.7	116.6	108.3	135.2	20.8
8/15/2018	38.1	57.6	73.8	135.2	20.8
8/22/2018	43.2	57.6	111.7	54.4	46.3
9/22/2018	60.7	57.6	62.8	11.9	11.5

Zooplankton diel migration pattern (Night-Day Individuals*L^{-1})

Datetime	Zoo2018	Zoo2017	Zoo2016	Zoo2015	Zoo2014
6/20/2018	10.0	6.8	25.6	-0.6	-5.1
7/18/2018	4.4	18.3	32.1	-40.8	6.5
8/1/2018	2.4	6.5	-14.7	-3.6	4.6
8/9/2018	0.4	6.5	-0.9	45.7	13.4
8/15/2018	3.8	9.3	6.6	45.7	13.4
8/22/2018	-2.6	9.3	5.5	-32.3	14.9
9/22/2018	2.3		32.2	3.9	3.7

Zooplankton genus proportion of total biomass (community composition)

		Daphnia	Diaptomus	Diacyclops	Bosmina	Holopedium
20-Jun-18	2018	0.58	0.08	0.32	0.01	0.01
	2014-2017	0.46	0.06	0.45	0.00	0.03
18-Jul-18	2018	0.43	0.18	0.30	0.01	0.08
	2014-2017	0.58	0.10	0.23	0.01	0.07
1-Aug-18	2018	0.69	0.08	0.18	0.01	0.05
	2014-2017	0.74	0.12	0.09	0.01	0.03
9-Aug-18	2018	0.63	0.13	0.20	0.02	0.02
	2014-2017	0.60	0.15	0.11	0.14	0.00
15-Aug-18	2018	0.79	0.06	0.14	0.01	0.00
	2014-2017	0.55	0.16	0.13	0.16	0.00
22-Aug-18	2018	0.76	0.14	0.09	0.01	0.00
	2014-2017	0.68	0.09	0.19	0.04	0.00
22-Sep-18	2018	0.84	0.10	0.06	0.00	0.00
	2014-2017	0.68	0.17	0.07	0.08	0.00

Yreka $PM_{2.5}$ Data is available at https://www.epa.gov/outdoor-air-quality-data/download-daily-data
